# Supplementary material for: Survival in Kidney and Bladder Cancers in Four Nordic Countries through a Half Century
Source: Cancers (Basel). 2023 May 16;15(10):2782. doi: 10.3390/cancers15102782 (PMC10216153; doi:10.3390/cancers15102782)
Supplement: Supplementary file 1 [file cancers-15-02782-s001.zip › cancers-2300931-supplementary.pdf]

SURVIVAL IN KIDNEY AND BLADDER CANCERS IN FOUR NORDIC COUNTRIES THROUGH A HALF CENTURY

Filip Tichanek, Asta Försti, Akseli Hemminki, Otto Hemminki, Kari Hemminki

# Supplementary Information

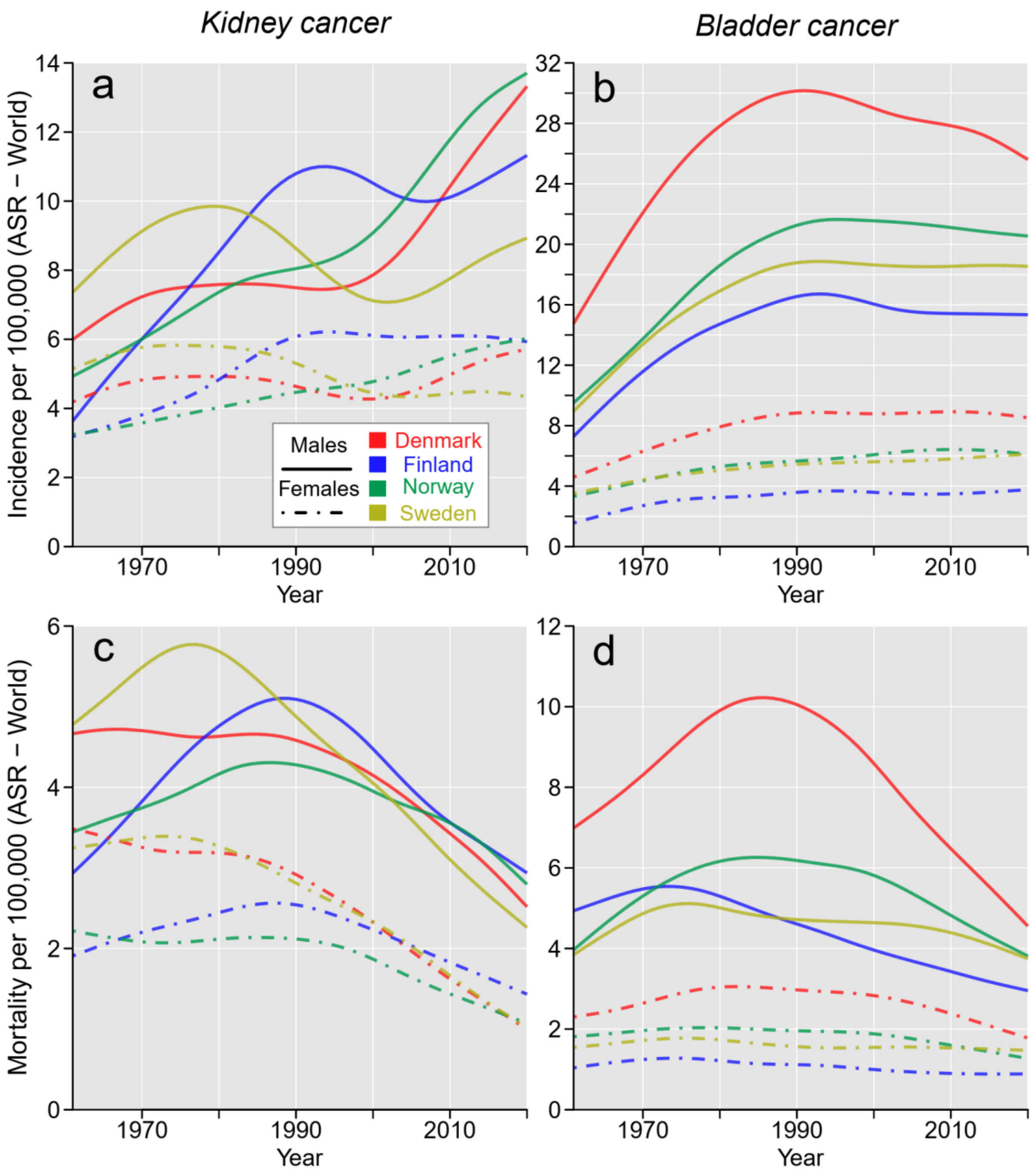


**Figure S1**. Incidence (a,b) and mortality (c,d) in kidney (a,c) and bladder (b,d) cancers from 1961 to 2020 in Denmark, Finland, Norway, and Sweden, separately for males and females. For a graphic presentation, the lines were smoothed by the cubic smoothing spline using the R function ‘smooth.spline’ with a smoothing parameter (‘spar’) of 0.3 and with 12 knots.

**Table S1**. 1-year and 5-year relative survival (95% confidence interval) in kidney and bladder cancers from 1971 to 2020. *Significant increase between the marked and the next period (nonoverlapping 95% CIs).

| Male cancers | | | | | Female cancers | | | |
|---|---|---|---|---|---|---|---|---|
| **Kidney 1-y** | Denmark | Finland | Norway | Sweden | Denmark | Finland | Norway | Sweden |
| 1971–1975 | 47.9 (44.4–51.7) | 55.8 (51.0–61.1) | 53.5 (49.4–58.0) | 49.5 (47.4–51.7)* | 49.4 (45.6–53.5) | 56.9 (52.3–62.0) | 60.7 (56.1–65.6) | 54.9 (52.5–57.3)* |
| 1976–1980 | 51.4 (48.2–54.9) | 53.7 (49.4–58.3) | 58.1 (54.5–62.1) | 58.8 (56.6–61.2) | 47.6 (44.3–51.1) | 59.3 (55.3–63.7) | 59.2 (55.2–63.5) | 60.9 (58.4–63.5) |
| 1981–1985 | 51.5 (48.5–54.6) | 56.7 (53.4–60.2)* | 58.0 (54.9–61.4) | 62.5 (60.3–64.7) | 53.5 (50.3–57.0) | 63.7 (60.6–67.0)* | 63.0 (59.4–66.9) | 64.0 (61.7–66.4) |
| 1986–1990 | 55.7 (52.7–58.8) | 66.0 (63.1–69.1)* | 61.3 (58.4–64.4)* | 66.2 (64.2–68.3)* | 52.8 (49.7–56.1) | 70.6 (67.9–73.3) | 66.4 (63.1–69.8) | 66.4 (64.1–68.6)* |
| 1991–1995 | 57.4 (54.5–60.4)* | 72.6 (70.1–75.2) | 67.5 (64.7–70.4) | 71.8 (69.9–73.8) | 56.7 (53.6–60.1) | 71.7 (69.3–74.2)* | 70.4 (67.3–73.6) | 73.7 (71.7–75.9) |
| 1996–2000 | 63.9 (61.2–66.8) | 73.2 (70.9–75.6) | 67.5 (64.9–70.3)* | 71.9 (69.9–73.9)* | 59.3 (56.2–62.5) | 77.5 (75.3–79.9) | 71.1 (68.1–74.3)* | 74.8 (72.7–77.0) |
| 2001–2005 | 64.8 (62.3–67.4)* | 75.7 (73.5–78.0) | 75.4 (73.2–77.7)* | 77.7 (75.9–79.6)* | 63.3 (60.2–66.5)* | 77.2 (75.1–79.4)* | 77.3 (74.6–79.9)* | 77.3 (75.2–79.4)* |
| 2006–2010 | 73.8 (71.8–76.0)* | 76.7 (74.7–78.8) | 80.6 (78.7–82.5)* | 84.3 (82.8–85.8)* | 74.5 (72.0–77.1)* | 81.5 (79.6–83.4) | 83.8 (81.6–86.1) | 86.0 (84.4–87.8) |
| 2011–2015 | 83.6 (82.0–85.3)* | 79.8 (78.0–81.5)* | 85.5 (83.8–87.2)* | 89.7 (88.5–90.9) | 83.2 (81.1–85.2)* | 83.2 (81.5–85.0) | 86.6 (84.6–88.7)* | 89.2 (87.7–90.6) |
| 2016–2020 | 88.1 (86.8–89.5) | 85.7 (84.3–87.1) | 90.2 (88.9–91.5) | 91.3 (90.2–92.3) | 88.2 (86.5–90.0) | 84.6 (82.9–86.3) | 90.9 (89.1–92.7) | 90.9 (89.6–92.3) |
| **Bladder 1-y** | | | | | | | | |
| 1971–1975 | 76.3 (74.8–77.8)* | 77.4 (74.7–80.0) | 78.9 (77.0–80.9)* | 79.0 (77.7–80.4)* | 71.3 (68.8–74.0) | 74.7 (70.7–78.9) | 71.8 (68.7–75.1) | 77.7 (75.8–79.7) |
| 1976–1980 | 79.9 (78.6–81.2) | 81.7 (79.7–83.7) | 83.9 (82.4–85.5) | 83.1 (82.0–84.3)* | 72.0 (69.8–74.3) | 76.7 (73.5–80.0) | 74.8 (72.0–77.6) | 78.5 (76.5–80.5) |
| 1981–1985 | 81.8 (80.7–83.0) | 84.8 (83.0–86.5) | 85.5 (84.1–86.9) | 86.0 (85.0–87.0) | 72.8 (70.7–74.9) | 81.2 (78.3–84.3) | 78.9 (76.4–81.5) | 82.2 (80.5–83.9) |
| 1986–1990 | 83.2 (82.1–84.3) | 86.6 (85.0–88.1) | 86.4 (85.2–87.7)* | 87.3 (86.4–88.2) | 76.5 (74.7–78.4) | 82.3 (79.6–85.0) | 80.3 (77.9–82.8) | 81.5 (79.9–83.1)* |
| 1991–1995 | 85.3 (84.3–86.3) | 87.9 (86.6–89.3) | 89.3 (88.1–90.4) | 88.5 (87.6–89.3) | 78.1 (76.4–80.0) | 81.7 (79.3–84.2) | 81.0 (78.8–83.3) | 84.7 (83.2–86.2) |
| 1996–2000 | 86.0 (85.1–87.0) | 89.1 (87.8–90.4) | 87.9 (86.8–89.1) | 88.4 (87.6–89.2) | 78.0 (76.2–79.8) | 85.9 (83.6–88.1) | 81.5 (79.3–83.8) | 84.2 (82.7–85.6) |
| 2001–2005 | 87.1 (86.1–88.0) | 89.6 (88.4–90.9) | 88.9 (87.9–89.9) | 88.5 (87.7–89.3) | 79.0 (77.3–80.7) | 83.8 (81.4–86.3) | 81.1 (79.0–83.3) | 83.5 (82.0–84.9) |
| 2006–2010 | 88.1 (87.2–88.9)* | 89.0 (87.9–90.1) | 89.2 (88.1–90.2) | 88.4 (87.6–89.2)* | 80.5 (78.8–82.2)* | 87.1 (85.1–89.1) | 84.1 (82.2–86.1) | 85.4 (84.0–86.8) |
| 2011–2015 | 90.3 (89.5–91.0) | 90.2 (89.3–91.2) | 90.9 (90.0–91.8) | 90.9 (90.2–91.5)* | 85.3 (84.0–86.7) | 83.9 (81.6–86.2) | 85.4 (83.7–87.2) | 85.1 (83.7–86.5) |
| 2016–2020 | 91.1 (90.4–91.8) | 90.6 (89.7–91.5) | 91.5 (90.6–92.3) | 92.4 (91.8–92.9) | 86.2 (84.8–87.6) | 85.8 (84.0–87.6) | 87.5 (85.9–89.1) | 86.9 (85.7–88.1) |
| **Kidney 5-y** | | | | | | | | |
| 1971–1975 | 27.0 (23.6–30.9) | 28.4 (23.5–34.3) | 33.1 (29.0–37.7) | 31.5 (29.4–33.8)* | 29.7 (26.0–33.9) | 34.6 (30.0–40.0) | 38.9 (34.1–44.5) | 38.0 (35.5–40.5) |
| 1976–1980 | 30.2 (26.8–34.0) | 29.8 (25.9–34.3) | 36.9 (32.7–41.7) | 38.1 (35.4–41.0) | 29.3 (26.0–32.9) | 34.3 (30.3–38.8)* | 38.5 (34.2–43.3) | 40.9 (38.2–43.8) |
| 1981–1985 | 30.4 (27.3–33.9) | 34.3 (30.7–38.4)* | 39.3 (35.7–43.2) | 41.3 (38.7–44.1) | 34.8 (31.5–38.5) | 43.2 (39.7–46.9)* | 42.6 (38.6–47.0) | 44.2 (41.6–46.9) |
| 1986–1990 | 34.9 (31.6–38.5) | 44.9 (41.3–48.9)* | 40.9 (37.5–44.6)* | 45.0 (42.5–47.6) | 35.4 (32.2–39.1) | 51.8 (48.5–55.3) | 49.8 (46.0–53.8) | 45.2 (42.7–47.9)* |
| 1991–1995 | 37.9 (34.7–41.5) | 54.1 (50.7–57.7) | 49.1 (45.6–52.8) | 49.6 (47.1–52.3) | 38.2 (34.8–41.8) | 57.2 (54.2–60.3) | 54.5 (50.8–58.5) | 55.3 (52.7–58.0) |
| 1996–2000 | 39.9 (36.5–43.5) | 58.0 (54.7–61.4) | 47.9 (44.8–51.4)* | 51.9 (49.4–54.6)* | 40.2 (36.9–43.8) | 61.4 (58.5–64.5) | 52.7 (49.1–56.6)* | 56.9 (54.3–59.8) |
| 2001–2005 | 45.9 (42.9–49.2)* | 58.4 (55.5–61.5) | 59.0 (56.0–62.1) | 59.7 (57.1–62.3)* | 45.0 (41.6–48.8)* | 63.5 (60.7–66.3) | 62.5 (59.1–66.0)* | 61.2 (58.5–64.0)* |
| 2006–2010 | 58.1 (55.4–61.0)* | 61.2 (58.6–63.9) | 64.0 (61.3–66.8)* | 68.7 (66.4–71.1)* | 58.5 (55.3–61.8)* | 67.0 (64.4–69.6) | 71.3 (68.2–74.5) | 70.9 (68.4–73.5)* |
| 2011–2015 | 68.7 (66.2–71.3)* | 64.8 (62.4–67.2)* | 74.7 (72.2–77.3) | 74.7 (72.7–76.8)* | 68.7 (65.8–71.7) | 71.1 (68.8–73.6) | 77.2 (74.3–80.2) | 76.6 (74.4–78.9) |
| 2016–2020 | 74.5 (72.2–76.8) | 71.2 (69.0–73.5) | 78.9 (76.6–81.2) | 79.0 (77.1–81.0) | 74.0 (71.3–76.9) | 74.0 (71.7–76.3) | 80.0 (77.1–83.1) | 78.6 (76.4–80.8) |
| **Bladder 5-y** | | | | | | | | |
| 1971–1975 | 54.9 (52.8–57.1)* | 50.2 (46.7–54.0) | 58.9 (56.1–61.8)* | 62.3 (60.3–64.3)* | 49.7 (46.5–53.1) | 48.6 (43.6–54.0)* | 51.2 (47.2–55.5) | 62.1 (59.5–64.9) |
| 1976–1980 | 59.7 (57.8–61.6) | 55.8 (52.8–58.9)* | 64.5 (62.1–67.0) | 68.9 (67.1–70.7) | 53.7 (50.9–56.7) | 58.4 (54.4–62.7) | 57.4 (53.9–61.1) | 63.7 (61.1–66.5) |
| 1981–1985 | 62.9 (61.1–64.7) | 66.5 (63.7–69.4) | 67.0 (64.7–69.4) | 69.5 (67.8–71.1)* | 54.2 (51.6–56.9)* | 64.2 (60.1–68.6) | 60.9 (57.7–64.4) | 65.6 (63.3–68.1) |
| 1986–1990 | 64.9 (63.2–66.7)* | 70.0 (67.4–72.7) | 70.5 (68.3–72.7) | 73.0 (71.5–74.5) | 60.0 (57.5–62.5) | 64.0 (60.2–68.0) | 64.5 (61.3–67.9) | 67.6 (65.4–69.9) |
| 1991–1995 | 68.7 (67.0–70.4) | 74.5 (72.1–76.9) | 73.5 (71.5–75.5) | 73.8 (72.4–75.2) | 63.2 (60.8–65.7) | 66.4 (63.0–69.9) | 64.7 (61.6–67.9) | 68.9 (66.7–71.0) |
| 1996–2000 | 71.0 (69.4–72.6) | 74.8 (72.6–77.1) | 72.9 (70.9–74.8) | 74.3 (72.9–75.7) | 61.6 (59.2–64.0) | 72.9 (69.6–76.4) | 68.8 (65.8–71.9) | 70.2 (68.1–72.2) |
| 2001–2005 | 73.4 (71.8–74.9) | 75.7 (73.6–77.9) | 75.4 (73.6–77.2) | 76.0 (74.7–77.3) | 64.5 (62.2–66.8) | 71.4 (68.2–74.8) | 67.1 (64.3–70.0) | 69.8 (67.8–71.9) |
| 2006–2010 | 75.5 (74.0–76.9)* | 77.5 (75.7–79.4) | 76.3 (74.6–78.1) | 75.4 (74.2–76.7)* | 66.5 (64.3–68.8)* | 75.4 (72.3–78.6) | 70.5 (67.8–73.4) | 72.1 (70.1–74.1) |
| 2011–2015 | 78.6 (77.3–79.9) | 78.6 (76.9–80.3) | 79.5 (78.0–81.1) | 79.3 (78.2–80.5)* | 72.1 (70.1–74.2) | 72.5 (69.4–75.8) | 71.8 (69.1–74.6) | 72.6 (70.7–74.5) |
| 2016–2020 | 81.0 (79.7–82.2) | 78.9 (77.3–80.5) | 81.1 (79.7–82.6) | 81.6 (80.6–82.6) | 74.0 (72.0–76.1) | 74.8 (72.1–77.7) | 75.6 (73.2–78.1) | 76.1 (74.4–77.9) |

**Table S2**. 5/1-year conditional survival in kidney and bladder cancers from 1971 to 2020.

| Male cancers | | | | | Female cancers | | | |
|---|---|---|---|---|---|---|---|---|
| **Kidney** | Denmark | Finland | Norway | Sweden | Denmark | Finland | Norway | Sweden |
| 1971–1975 | 56.4 | 50.9 | 61.9 | 63.6 | 60.1 | 60.8 | 64.1 | 69.2 |
| 1976–1980 | 58.8 | 55.5 | 63.5 | 64.8 | 61.6 | 57.8 | 65.0 | 67.2 |
| 1981–1985 | 59.0 | 60.5 | 67.8 | 66.1 | 65.0 | 67.8 | 67.6 | 69.1 |
| 1986–1990 | 62.7 | 68.0 | 66.7 | 68.0 | 67.0 | 73.4 | 75.0 | 68.1 |
| 1991–1995 | 66.0 | 74.5 | 72.7 | 69.1 | 67.4 | 79.8 | 77.4 | 75.0 |
| 1996–2000 | 62.4 | 79.2 | 71.0 | 72.2 | 67.8 | 79.2 | 74.1 | 76.1 |
| 2001–2005 | 70.8 | 77.1 | 78.2 | 76.8 | 71.1 | 82.3 | 80.9 | 79.2 |
| 2006–2010 | 78.7 | 79.8 | 79.4 | 81.5 | 78.5 | 82.2 | 85.1 | 82.4 |
| 2011–2015 | 82.2 | 81.2 | 87.4 | 83.3 | 82.6 | 85.5 | 89.1 | 85.9 |
| 2016–2020 | 84.6 | 83.1 | 87.5 | 86.5 | 83.9 | 87.5 | 88.0 | 86.5 |
| **Bladder** | | | | | | | | |
| 1971–1975 | 72.0 | 64.9 | 74.7 | 78.9 | 69.7 | 65.1 | 71.3 | 79.9 |
| 1976–1980 | 74.7 | 68.3 | 76.9 | 82.9 | 74.6 | 76.1 | 76.7 | 81.1 |
| 1981–1985 | 76.9 | 78.4 | 78.4 | 80.8 | 74.5 | 79.1 | 77.2 | 79.8 |
| 1986–1990 | 78.0 | 80.8 | 81.6 | 83.6 | 78.4 | 77.8 | 80.3 | 82.9 |
| 1991–1995 | 80.5 | 84.8 | 82.3 | 83.4 | 80.9 | 81.3 | 79.9 | 81.3 |
| 1996–2000 | 82.6 | 84.0 | 82.9 | 84.0 | 79.0 | 84.9 | 84.4 | 83.4 |
| 2001–2005 | 84.3 | 84.5 | 84.8 | 85.9 | 81.6 | 85.2 | 82.7 | 83.6 |
| 2006–2010 | 85.7 | 87.1 | 85.5 | 85.3 | 82.6 | 86.6 | 83.8 | 84.4 |
| 2011–2015 | 87.0 | 87.1 | 87.5 | 87.2 | 84.5 | 86.4 | 84.1 | 85.3 |
| 2016–2020 | 88.9 | 87.1 | 88.6 | 88.3 | 85.8 | 87.2 | 86.4 | 87.6 |
